# How Explainable Artificial Intelligence Can Increase or Decrease Clinicians’ Trust in AI Applications in Health Care: Systematic Review

**DOI:** 10.2196/53207

**Published:** 2024-10-30

**Authors:** Rikard Rosenbacke, Åsa Melhus, Martin McKee, David Stuckler

**Affiliations:** 1 Centre for Corporate Governance Department of Accounting Copenhagen Business School Frederiksberg Denmark; 2 Department of Medical Sciences Clinical Microbiology Uppsala University Uppsala Sweden; 3 European Observatory on Health Systems and Policies London School of Hygiene & Tropical Medicine London United Kingdom; 4 Department of Social and Political Sciences Bocconi University Milano Italy

**Keywords:** explainable artificial intelligence, XAI, trustworthy AI, clinician trust, affect-based measures, cognitive measures, clinical use, clinical decision-making, clinical informatics

## Abstract

**Background:**

Artificial intelligence (AI) has significant potential in clinical practice. However, its “black box” nature can lead clinicians to question its value. The challenge is to create sufficient trust for clinicians to feel comfortable using AI, but not so much that they defer to it even when it produces results that conflict with their clinical judgment in ways that lead to incorrect decisions. Explainable AI (XAI) aims to address this by providing explanations of how AI algorithms reach their conclusions. However, it remains unclear whether such explanations foster an appropriate degree of trust to ensure the optimal use of AI in clinical practice.

**Objective:**

This study aims to systematically review and synthesize empirical evidence on the impact of XAI on clinicians’ trust in AI-driven clinical decision-making.

**Methods:**

A systematic review was conducted in accordance with PRISMA (Preferred Reporting Items for Systematic Reviews and Meta-Analyses) guidelines, searching PubMed and Web of Science databases. Studies were included if they empirically measured the impact of XAI on clinicians’ trust using cognition- or affect-based measures. Out of 778 articles screened, 10 met the inclusion criteria. We assessed the risk of bias using standard tools appropriate to the methodology of each paper.

**Results:**

The risk of bias in all papers was moderate or moderate to high. All included studies operationalized trust primarily through cognitive-based definitions, with 2 also incorporating affect-based measures. Out of these, 5 studies reported that XAI increased clinicians’ trust compared with standard AI, particularly when the explanations were clear, concise, and relevant to clinical practice. In addition, 3 studies found no significant effect of XAI on trust, and the presence of explanations does not automatically improve trust. Notably, 2 studies highlighted that XAI could either enhance or diminish trust, depending on the complexity and coherence of the provided explanations. The majority of studies suggest that XAI has the potential to enhance clinicians’ trust in recommendations generated by AI. However, complex or contradictory explanations can undermine this trust. More critically, trust in AI is not inherently beneficial, as AI recommendations are not infallible. These findings underscore the nuanced role of explanation quality and suggest that trust can be modulated through the careful design of XAI systems.

**Conclusions:**

Excessive trust in incorrect advice generated by AI can adversely impact clinical accuracy, just as can happen when correct advice is distrusted. Future research should focus on refining both cognitive and affect-based measures of trust and on developing strategies to achieve an appropriate balance in terms of trust, preventing both blind trust and undue skepticism. Optimizing trust in AI systems is essential for their effective integration into clinical practice.

## Introduction

Artificial intelligence (AI) is increasingly being promoted as a means to transform health care. AI can enhance clinical decision-making, reduce medical errors, and improve patient outcomes [1,2]. Yet, to realize its full potential in health care, clinicians must trust it and be comfortable with its outputs [3]. Establishing and maintaining trust is challenging, especially in light of growing warnings from some leading AI experts about its potential risks to society [4].

Currently, there is a dearth of studies on how to increase trust in AI among clinicians. In a recent systematic review on trust in AI, it was observed that transparency is critical for fostering trust among decision makers [5]. To increase transparency and, thus, trust in AI, it has been proposed that measures should be added to its predictions to make the models more transparent and explainable to human users [6]. So-called explainable AI (XAI) can be considered to fall within several categories: (1) “local” (specific) explanations of an individual prediction [7], (2) “global” explanations presenting the model’s general logic [8], (3) “counterfactual” explanations indicating a threshold at which the algorithm could change its recommendations, (4) confidence explanations, indicating the probability that the prediction is correct [9]; and (5) example-based, where the AI justifies its decision by providing examples that have similar characteristics from the same dataset [10].

Trust is a complex concept that has been explored in a range of disciplines, including philosophy, economics, sociology, and psychology [11-15], with a recent review by one of us [16] noting how little interaction exists between these disciplinary perspectives. Here, we rely on psychological models, which we consider to be particularly helpful in this context. In a dual theory developed by Kahneman [17], 2 main ways of thinking exist. The first is quick and based on gut feelings or intuition, whereas the second is slower, taking a more thoughtful and reasoning approach. Trust forms a mental picture of another person or a system, and when trying to untangle all its intricacies, it is practically impossible to use only rational thought. Consequently, the decision to trust someone or something like an AI tool or a physician is often derived from an instinctive judgment or intuition. In this model, trust is viewed as a decision-making shortcut, enabling the decision maker to select information while ignoring other information to simplify a complex decision [18]. Applied to empirical research, Madsen et al [19] describe these 2 broad approaches as cognition-based trust and affect-based trust, terms that we will use in this study.

A series of recent reviews have examined XAI from a trusted perspective. However, partly reflecting the speed of development of the field, these do not include the most recent empirical evidence from clinical settings, although they did consistently speculate that XAI could increase users’ trust and thus the intention to use AI tools [20,21], as well as enhance confidence in decisions and thus, the trust of clinicians [22,23]. None of these studies differentiated between varying trust measures or health care domains.

To fill this gap, we performed a systematic review of empirical evidence on the impact of XAI on clinicians’ trust. In addition, we categorized and differentiated studies according to which type of trust measure they used, cognition- or affect-based trust, as well as types of medical data used (imaging vs tabular formats).

## Methods

### Search Strategy

A total of 2 authors (RR and DS) performed a systematic review in accordance with the PRISMA (Preferred Reporting Items for Systematic Reviews and Meta-Analyses) guidelines [24]. On March 23, 2023, we searched the title and abstract fields of PubMed and recognized that the topic would be covered by a wide range of disciplines; hence, we also used the Web of Science database. We searched for published articles on XAI and trust within health care. Our initial reading revealed the use of many words that conveyed some aspect of what we might consider “trust.” In light of this work and the many different conceptions of trust [25], we intentionally used a broad search strategy without specifying trust and its alternative variants (such as confidence, intention to use, etc) to avoid the risk of “type-2 errors” whereby relevant articles that should have been included were omitted.

We operationalized XAI and health care using a range of keyword permutations adapted to each database (full strategy in Multimedia Appendix 1).

### Inclusion and Exclusion Criteria

We applied a range of inclusion and exclusion criteria. Articles were included if they (1) measured trust (and related terms) as an outcome, (2) used XAI as an intervention or exposure, (3) used machine learning (ML) in the underlying AI model, (4) were empirical studies, and (5) were carried out by practicing clinicians. Articles were excluded if they were (1) reviews, commentaries, reports of methodology, or conceptual papers or (2) not applied in a health care setting from a clinician’s perspective. Furthermore, 2 reviewers, RR and DS, performed the screening, and any disputes were resolved against these prespecified criteria and with a third reviewer (ÅM).

### Extraction and Analysis

We extracted from each included study the following data: author, year of publication, country, health care domain, discipline behind the study, image versus tabular data input, study design and setting, clinical or experimental setting, sample size, intervention or exposure of interest, outcome measures, study results, and conclusions. Data were entered into a Microsoft Excel spreadsheet for analysis. RR extracted the data using the preestablished data entry format, with verification by DS to ensure consistency. We disaggregated the analysis by trust dimensions (cognitive versus affect-based) and by type of data evaluated (image versus tabular data). We also assessed each paper for risk of bias, using either the Cochrane Risk of Bias 2 (RoB 2) or Risk of Bias in Non-randomized Studies of Interventions (ROBINS-I) tool.

## Results

### Overview of Search Results

**Figure 1:**
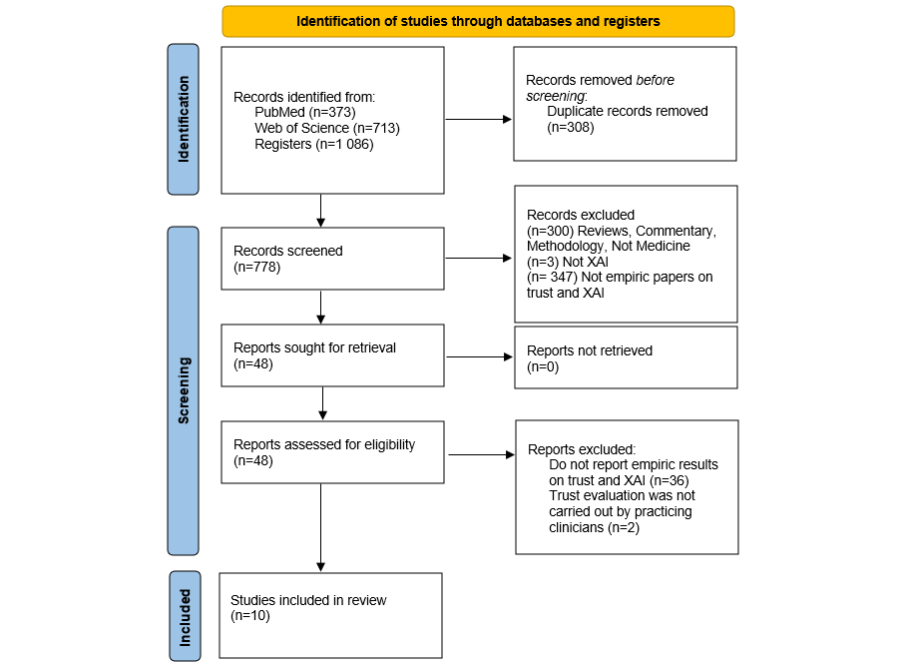
PRISMA (Preferred Reporting Items for Systematic Reviews and Meta-Analyses) flow chart. XAI: explainable artificial intelligence.

Our initial search identified 373 publications in PubMed and 713 publications in Web of Science, 308 of which were duplicates, leaving 778 for the screening and eligibility stages. We excluded 300 records since they were reviews, commentaries, methodological reports, conceptual papers, or not related to the health care sector. A total of 83 papers did not study XAI, and 347 were not empirical studies with trust as an outcome and explanations as an intervention. This left 48, all of which were successfully retrieved. We excluded another 38 studies when reviewing the full text as they did not measure trust or XAI empirically, or the evaluation was not carried out by practicing clinicians. This yielded 10 articles for the final review (Figure 1) [26-35].

The publications were imported into Zotero (Corporation for Digital Scholarship) reference management software. The PRISMA flow diagram of our review is shown in Figure 1 (PRISMA checklist provided in Multimedia Appendix 2).

### Characteristics of Included Studies

Table 1 provides a summary of the final studies. There was a clear increase in papers on trust and XAI in health care during 2022; 70% (7/10) were published between 2022 and the end of the inclusion period on March 23, 2023.

**Table 1:** Summary of the extracted studies.

Title	Authors (Year) Country	Study discipline	Respondents (Sample size, n)	Health care domain	Tabular or Image	Description of intervention	Trust measurement	Trust improvement
As if sand were stone. New concepts and metrics to probe the ground on which to build trustable AI	Cabitza et al [26] (2020) Italy	Computer science, Orthopedic and biomedicine	Physician (13)	Radiology	Image	Measure radiologists’ confidence score as a marker for trust	Quantitative confidence score, 6-grade scale.	No effect
Doctor’s dilemma: Evaluating an explainable subtractive spatial lightweight convolutional neural network for brain tumor diagnosis	Kumar et al [27] (2021) India	Computer science	Physicians (10)	Brain tumor	Image	Building an explainable deep learning model to reduce complexity in MR classifications.	Quantitative doctor survey using 5-grade Likert Scale.	Increased trust
Does AI explainability affect physicians' intention to use AI?	Liu et al (2022) [35] Taiwan	Medical research, cardiology, pediatrics	Physicians (295)	—^a^	Image	Comparing intention to use XAI vs AI	Quantitative survey using a 5-grade scale.	Increased trust
Explainable recommendation: when design meets trust calibration.	Naiseh et al [28] (2021) United Kingdom	Computer science	Physicians and pharmacists (24)	Oncology	Tabular	Involved physicians and pharmacists in think-aloud study and codesign to identify potential trust calibration errors	Qualitative interviews analyzed using content analysis.	Varied, depending on factors such as the form of explanation
How the different explanation classes impact trust calibration: The case of clinical decision support systems	Naiseh et al [29] (2023) United Kingdom	Computer science	Physicians and pharmacists (41)	Chemotherapy	Tabular	Trust calibration for 4 XAI classes (counterfactuals, example-based, global and local explanations) vs no explanations	Quantitative self-reporting cognitive-based trust using a 5-grade scale and qualitative interviews were coded.	Varied, depending on factors such as the form of explanation
Interpretable clinical time-series modeling with intelligent feature selection for early prediction of antimicrobial multidrug resistance	Martínez-Agüero et al [34] (2022) Spain	Computer science and Intensive care department for validation	Clinicians (no specification)	Antibiotic resistance	Tabular	SHAP explanations for predictors to provide clinicians with explanations in natural language	Qualitative, where clinicians self-report.	Increased trust
Nontask expert physicians benefit from correct explainable AI advice when reviewing X-rays.	Gaube et al [33] (2023) United States and Canada	Medicine, psychology, and computer science	Internal or emergency medicine physicians and radiologists (223)	Radiology	Image	Visible annotation on the X-ray done by human or XAI	Quantitative self-reporting using 7-grade scale.	No effect
The explainability paradox: Challenges for XAI in digital pathology	Evans, et al [30] (2022)	Computer science and biomedicine	Board-certified pathologists and professionals in pathology or neuropathology (6+25)	Pathology	Image	Saliency maps to explain predictions through visualizations	Quantitative self-reporting using 7-grade scale. Qualitative semistructured interviews.	Increased trust
Trustworthy AI explanations as an interface in medical diagnostic systems	Kaur et al [31] (2022) United States	Computer science	Physicians (2)	Breast cancer prediction	Image	Involved physicians evaluate 3 different systems and rate them “Trustworthy Explainability Acceptance.”	Quantitative, trust is calculated using both impression and confidence.	Developed framework to measure trust. No effect identified
UK reporting radiographers’ perceptions of AI in radiographic image interpretation current perspectives and future developments	Rainey et al [32] (2022) United Kingdom	Health science, radiography, and computer science	Radiographers (86)	Radiography	Image	—	Quantitative self-reporting using 10-grade scale.	Increased trust

^a^Not applicable.

The studies displayed marked heterogeneity in methods, disciplinary collaboration, and perspectives of trust. All but 1 involved computer scientists; 4 were conducted solely by computer scientists without involvement by experts with a medical background, and the remaining 5 involved collaborations between medical experts and computer scientists. The inputs to the AI tools were medical imaging or tabular data formats. The risk of bias in each study is reported in Multimedia Appendix 3. In all studies, the risk of bias was moderate or moderate to high.

We begin by looking at studies of medical imaging and tabular data separately, providing an overview of the characteristics and results before moving on to talk about the different ways in which studies conceptualize or measure trust (as we found that this seemed to be a key consideration in interpreting studies’ results).

### Medical Imaging

Out of the 7 medical imaging studies reviewed, 4 (57%) identified a significant and positive association between the use of XAI and perceived trust, 1 study (14%) reached no clear conclusions, while 2 (29%) found limited or no significant impact.

A study by Liu et al [35] asked 295 physicians across 3 hospitals in Taiwan if explanations increased their trust in the algorithm and their propensity to use XAI compared with AI. They found that physicians were more inclined to trust and implement AI in clinical practice if they perceived the results as being more explainable or comprehensible. Similarly, a web-based experiment by Evans et al [30] surveyed trust levels among board-certified physicians in pathology or neuropathology in using XAI to interpret pathology images. The XAI instrument highlighted the areas in medical images that determined whether the prediction was made with high or low confidence. In addition, 70% agreed that their level of trust increased as a result of the explanations provided, while approximately 10% disagreed, and the rest were undecided.

A study by Cabitza et al [26] differentiated Gold Standard labels (categorizing cases as positive or negative) from Diamond Standard ones, where the reason for categorization was annotated and indicated confidence in the allocation. A total of 13 radiologists were then asked to evaluate images of knees. Confidence in the allocation was considered a proxy for trust, and there was no association between confidence and accuracy. Gaube et al [33] conducted a qualitative investigation of 117 clinical residents or practicing emergency medicine physicians and 106 radiologists. They reported that explanations had little or no significant impact on the trust and the perceived usefulness of AI. The participants were shown x-rays with and without annotations as explanations. Internal and emergency medicine physicians (IM/EM), who lacked specialist training in radiology, achieved better diagnostic accuracy when provided with explanations (*P*_IM/EM_=.042), but there was no such benefit for radiologists (*P*_Radiology_=.12). In neither group did annotations have any meaningful effect on confidence in their own final diagnosis (*P*_IM/EM_=.280, *P*_Radiology_=.202). The authors did not find convincing evidence for either algorithmic appreciation (a tendency to trust algorithms) or algorithmic aversion (a tendency not to trust algorithms).

### Tabular Data

The 3 studies using XAI techniques with tabular data found positive relationships between explanations of AI and perceived trust. However, in 2 of the studies, results varied, and the authors argued that an inappropriate use of explanations can induce under- or overtrust.

A qualitative study by Martinez-Aguero et al [34] investigated whether XAI, when compared with AI, increased trust among clinicians searching for multidrug-resistant bacteria in intensive care units. The authors concluded that both visual and textual explanations helped clinicians understand the model output and increased trust in the XAI. However, neither the number of respondents nor the instrument used to measure trust was clearly reported.

Naiseh et al [28] performed a qualitative study on the influence of XAI on the prescribing decisions of physicians and pharmacists in the field of oncology. For the trust, they used the terminology used by Chiou and Lee [36] of appropriate reliance. They initially performed semistructured interviews with 16 participants to understand how these providers engaged with 5 distinct types of explanations: local, global, counterfactual, example-based, and confidence-based. The authors coded the providers as exhibiting “high” or “low” trust only if this behavior was consistent across all 5 explanation types in the study. Although the physicians and pharmacists were generally favorable toward explanations, they exhibited a lack of trust and skepticism about XAI’s accuracy. They further identified two primary causes of errors in trust calibration: (1) skipping explanations or (2) misapplication of explanations. Skipping occurred when providers made decisions with AI without fully engaging with the accompanying explanations. This was due to (1) disinterest in understanding the explanation, (2) decision delays due to the explanation, and (3) perceived redundancy, complexity, or context irrelevance. Misapplication occurred when the providers misunderstood the explanations or simply sought after them to confirm their initial judgement. They then conducted codesign sessions with 8 participants. From these, they proposed enhancing XAI interface designs to help avoid skipping or misinterpreting explanations. The designs included active or cognitive engagement of decision-makers in the decision-making process, challenge of habitual actions in the XAI system by introducing alternative perspectives or recommendations that may not align with the clinical decision-maker’s previous experiences or assumptions, friction that requires the decision-maker to confirm their decision before it is implemented, and support consisting of training and learning opportunities for clinical decision-makers to enhance the understanding and usage of the system.

This same team studied 41 medical practitioners who were frequent users of clinical decision support systems [29]. They sought to develop interventions that would enable physicians to have an optimal level of trust (or reliance), as defined by the authors, in predictions by AI models and to avoid errors that might arise from excessive under- or overtrust. The clinicians used 4 different XAI classes (global, local, counterfactual, and example-based; their other study had included confidence-based), and the research group explored the clinicians’ experiences using semistructured interviews. A subsequent mixed methods study on chemotherapy prescriptions found differences in the trust generated by different explanations. Participants found example-based and counterfactual explanations more understandable than the others, but there were no differences in perceptions of technical competence, a view supported in semistructured interviews, largely because they were easier to comprehend. In addition, the researchers identified a potential for overreliance on AI, as providers were more inclined to accept AI recommendations when they were accompanied by explanations, although explanations did not help them identify incorrect recommendations. They made a series of suggestions as to how the interface design might be enhanced, although they also noted that it could be very difficult to incorporate the many different types of questions that users might ask. Some might seek very detailed explanations, while others could be deterred by the resulting cognitive overload. As the authors note, “long and redundant explanations make participants skip them.” Perhaps more fundamentally, several of those interviewed said that they would be reluctant to use this tool because of the high cognitive load involved in seeking to understand some decisions.

### Conceptualizing and Measuring Trust

The studies that were reviewed take 2 broad approaches to defining trust: cognition-based trust and affect-based trust [19]. The initial approach, cognition-based trust, revolves around the perceived clarity and technical ability of XAI, fundamentally grounded in rational analysis. On the other hand, affect-based trust encompasses emotional bonds and beliefs originating from previous experiences and sentiments towards AI, as opposed to logical deliberation. All 10 studies applied cognitive-based trust. However, 2 studies also investigated trust in terms of affect or emotions.

A total of 8 studies used quantitative surveys to measure trust, integrating them with qualitative interviews in 2 instances. The remaining 2 exclusively used qualitative interviews. We found marked heterogeneity in the questions used.

Naiseh et al [28,29] noted that explanations affected both cognitive and affect-based trust and could result in either overtrust or undertrust. In the 2021 study [28], they used qualitative think-aloud methods and suggested that 1 reason for users skipping or misapplying explanations could be that affect-based trust overrides cognitive and deliberate trust. A couple of years later, they published a new study [29] in which they investigated whether different XAI classes or methods increased or decreased cognitive-based trust. They found that some types of explanation could introduce a cognitive overreliance on the AI, but they questioned whether biases and affect-based trust also played roles.

## Discussion

### Principal Findings

We examined empirical evidence on the impact of explainable AI on physicians’ trust levels and intention to use AI. Out of the 10 studies included, 50% (5/10) reported that XAI increased trust, while 20% (2/10) observed both increased and decreased trust levels. Both overtrust and undertrust appeared to be modifiable by brief cognitive interventions to optimize trust [28,29]. In 2 studies (20%), no effects of XAI were shown, and one study (10%) did not reach any conclusions. Only small differences of no consequence were identified between studies using tabular data formats and image data.

Before interpreting these findings further, we must note several important limitations of our study’s search strategy. First, there is considerable heterogeneity in the use of the term “trust” and how it is operationalized in health care research. To avoid potentially missing important studies in our search, we adopted a conservative search strategy in which we did not specify trust as a keyword but rather manually searched for all papers, including a broad set of trust-related outcomes. Related to this, the rapid evolution of AI has been associated with conceptual confusion about its meaning. Several recent studies have sought to operationalize AI in markedly varying ways, drawing on technology, for example, which is not actually based on AI algorithms [37,38]. For clarity, we specifically constrained our search to AI algorithms, which used machine-learning techniques. Second, we used 2 main databases of peer-reviewed studies, PubMed and Web of Science. The former has broad coverage in medicine and social sciences but could potentially miss emerging studies in computer science, but Clarivate, which publishes Web of Science, notes that it has “Strongest coverage of natural sciences & engineering, computer science, materials sciences, patents, data sets” [39]. We do, however, accept that, in a rapidly developing field, we may have missed material in preprints or non–peer-reviewed conference papers. In addition, for coherence across platforms, we did not use MeSH (Medical Subject Headings) terms in PubMed, as they are not used in Web of Science, and we wanted to achieve consistency. The keyword “clinical” also may potentially have excluded studies in some clinical specialties. However, the vast number of potential specialist terms that could be used makes it virtually impossible to implement a wider strategy in practice. Finally, there has been extensive study of psychological biases in how decision makers, including clinicians, respond to new data and update previous beliefs in incorporating evidence to make decisions [17,40]. Studies by psychologists are needed to evaluate the role these biases (including but not limited to default bias and confirmation bias) play in medical decision-making when using XAI.

A series of limitations were also identified in the included studies. Generally, the study designs widely varied, from qualitative investigations to experimental quantitative studies, making it difficult to draw direct comparisons. However, we have sought to the extent possible to identify emerging themes and patterns across tabular and visual XAI applications, as well as a series of methodological limitations to address in future studies. In addition, the relatively low number of studies (n=10) limits generalizability to other populations and settings. Another limitation present in several studies was the weak reporting of trust measurement instruments, as well as the number of respondents, particularly in qualitative studies. Few studies have reported the validity of the underlying XAI algorithm, which could also alter health care providers’ engagement and trust in XAI technologies. Future research should seek to improve the reporting of this necessary information.

Although our review focused on how XAI impacted clinicians’ trust levels and intention to use this technology, a few additional observations are of interest. Gaube et al [33] found no difference in trust between experts and nonexperts but reported that the performance of nonexperts who drew upon XAI was superior in clinical practice. Future studies are needed not just to evaluate the impact of XAI on its adoption and trustworthiness but also its potential clinical efficacy. In this context, it is worth noticing that while all included studies offered explanations that could be added to AI predictions, the validity of those explanations has yet to be critically evaluated [41] It is unclear how XAI can overcome limitations inherent in clinical domains where mechanistic understanding is lacking. That is, XAI will likely struggle to explain what is currently unexplainable at the frontier of clinical medicine. This could potentially lead to explanations that, albeit perceived as trustworthy, are not founded on established clinical knowledge and instead are “misconceptions” by AI. The XAI explanations are still simplifications of the original AI model, and when the abstraction level is heightened, the granularity is usually reduced.

This review also points to the need to understand how trust in XAI can be optimized rather than simply being evaluated in terms of increased or decreased with the help of different types of explanations. Clinical decision-making inevitably involves an element of judgment. While AI may be able to process more information than a human, humans may also be able to incorporate insights that are not included in algorithms [41]. Thus, the challenge is to achieve an appropriate level of trust in AI, neither too limited, in which case the clinician will be reluctant to use it, nor too extensive, as this may cause experienced clinicians to subordinate their own judgment to the AI outputs.

Yet, while it is apparent that neither blind trust nor blind distrust may be appropriate, it is unclear what an appropriate or optimal level of trust should be. None of the studies attempted to explore what this should be, which remains an important area for future research. However, the studies reviewed indicated that the levels of trust that health care providers place in AI depend on multiple clinically-relevant factors, including but not limited to the accuracy of the algorithm, the validation, and the potential impact on patients.

Our study also points to several further directions for future research. First, while the interdisciplinary literature featured prominent computer scientists and clinicians, there was a notable absence of psychologists. There is considerable scope to improve the appropriate uptake and adoption of AI by drawing upon evidence from the wider psychological literature on medical decision-making. One such framework is a dual process model, which integrates both cognitive and affect-based means of decision-making jointly. Kahneman [17] argues that the human mind uses 2 processes for decision-making: the fast thinking and intuitive process, including heuristics, biases, and cognitive shortcuts that recall affect-based trust, and the slow thinking and reasoning process that recalls cognitive-based trust. Furthermore, Thaler and Sunstein [42] have found that both these processes can be influenced (or nudged), especially the rapid thinking intuitive judgments. Brief cognitive interventions such as nudging have sometimes proven to be useful in health. The extant literature appears to incorporate mainly reasoning-based cognitive markers but misses out on intuitive and emotion-based processes for evaluating trust levels in emerging technologies.

### Conclusions

A majority of the included studies showed that XAI increases clinicians’ trust and intention to use AI; 2 of these studies showed that explanations could both increase and decrease trust and in 3 studies, explanations fell through or did not add any value. However, in health care, when AI tool incorporates associated explanations, they must avoid 2 common psychological pitfalls. First, they must be made sufficiently clear to avoid risks of blind distrust when physicians do not understand them. Second, they must avoid oversimplification and failing to disclose limitations in models that could lead to blind trust among physicians with an artificial level of clinical certainty. Explanations can both increase and decrease trust, and understanding the optimal level of trust in relation to the algorithm’s accuracy will be critical. When AI algorithms surpass physicians in terms of accuracy, the integration could be facilitated through means such as providing explanations. Yet, the provision of explanations is not a failsafe method to detect errors in the algorithms, as it might inadvertently foster excessive trust. How to find an optimal level of trust and how to best communicate AI to physicians will remain a defining health care challenge of our time.

## Appendix

Multimedia Appendix 1 Search strategy.

Multimedia Appendix 2 PRISMA (Preferred Reporting Items for Systematic Reviews and Meta-Analyses) checklist.

Multimedia Appendix 3 Assessment of risk of bias.

## Abbreviations

AI: artificial intelligence
IM/EM: internal and emergency medicine physicians
MeSH: Medical Subject Headings
ML: machine learning
PRISMA: Preferred Reporting Items for Systematic Reviews and Meta-Analyses
RoB 2: Risk of Bias 2
ROBINS-I: Risk of Bias in Non-randomized Studies of Interventions
XAI: explainable artificial intelligence
