# Supplementary material for: How Explainable Artificial Intelligence Can Increase or Decrease Clinicians’ Trust in AI Applications in Health Care: Systematic Review
Source: JMIR AI. 2024 Oct 30;3:e53207. doi: 10.2196/53207 (PMC11561425; doi:10.2196/53207)
Supplement: Multimedia Appendix 1 [file ai_v3i1e53207_app1.docx]

**Multimedia Appendix 1.** Search Strategy.

PubMed:

(XAI[Title/Abstract] OR "explainable artificial intelligence"[Title/Abstract] OR "explainable AI"[Title/Abstract]) AND (Healthcare[Title/Abstract] OR medical*[Title/Abstract] OR clinical*[Title/Abstract])

Web of Science:

#1 XAI OR "explainable artificial intelligence" OR "explainable AI" (Topic)

#2 healthcare OR medical* OR clinical* (Topic)

#1 AND #2
