# Supplementary material for: How Explainable Artificial Intelligence Can Increase or Decrease Clinicians’ Trust in AI Applications in Health Care: Systematic Review
Source: JMIR AI. 2024 Oct 30;3:e53207. doi: 10.2196/53207 (PMC11561425; doi:10.2196/53207)
Supplement: Multimedia Appendix 3 [file ai_v3i1e53207_app3.docx]

**Multimedia Appendix 2.** Assessment of risk of bias.

| Title | Authors (Year) Country | Tool | Risk | Reasons |
| --- | --- | --- | --- | --- |
| As if sand were stone. New concepts and metrics to probe the ground on which to build trustable AI | Cabitza *et al.* (2020) Italy | Cochrane Risk of Bias (RoB 2) | Moderate | Potential biases in labeling due to human judgment variability, potential deviations in rater performance, and how these issues are managed in the study's methodology. |
| Doctor's Dilemma: Evaluating an Explainable Subtractive Spatial Lightweight Convolutional Neural Network for Brain Tumor Diagnosis | Kumar *et al.* (2021) India | Cochrane Risk of Bias (RoB 2) | Moderate to high | Lack of representativeness, over-reliance on technical outcomes, and insufficient real-world validation of the model's performance and explainability. |
| Does AI explainability affect physicians' intention to use AI? | Liu *et al.* (2022) Taiwan | Risk of Bias in Non-randomized Studies of Interventions (ROBINS-I) | Moderate | Potential confounding factors, the use of convenience sampling, and the subjective nature of the self-reported outcomes. |
| Explainable recommendation: when design meets trust calibration. | Naiseh *et al.* (2021) UK | Cochrane Risk of Bias (RoB 2) | Moderate to high | Qualitative and non-randomized design, potential deviations due to participants' familiarity with AI, and subjective nature of the data collection and reporting processes |
| How the different explanation classes impact trust calibration: The case of clinical decision support systems | Naiseh e*t al.* (2023) UK | Risk of Bias in Non-randomized Studies of Interventions (ROBINS-I) | Moderate | While the study uses validated tools and consistent application of interventions, limitations such as lack of participant randomization, potential order effects, and reliance on self-reported measures could affect the robustness of the findings. |
| Interpretable clinical time-series modelling with intelligent feature selection for early prediction of antimicrobial multidrug resistance | Martinez-Aguero *et al.* (2022) Spain | Risk of Bias in Non-randomized Studies of Interventions (ROBINS-I) | Moderate to high | Potential confounding, selection bias, handling of missing data, and reliance on EHR data quality. |
| Non-task expert physicians benefit from correct explainable AI advice when reviewing X-rays. | Gaube *et al.* (2023) US/Canada | Risk of Bias in Non-randomized Studies of Interventions (ROBINS-I) | Moderate | Potential confounding factors, selection bias due to the recruitment strategy, and the use of self-reported measures that could affect validity. |
| The explainability paradox: Challenges for XAI in digital pathology | Evans, *et al.* (2022) | Risk of Bias in Non-randomized Studies of Interventions (ROBINS-I) | Moderate | Risk of selection bias, potential confounding due to uncontrolled participant variability, and measurement bias from self-reported data. |
| Trustworthy AI Explanations as an Interface in Medical Diagnostic Systems | Kaur *et al.* (2022) US | Cochrane Risk of Bias (RoB 2) | Moderate to high | Reliance on simulated expert profiles, the absence of detailed handling of missing data, and lack of a real-world clinical validation component. |
| UK reporting radiographers’ perceptions of AI in radiographic image interpretation Current perspectives and future developments | Rainey *et al.* (2022) UK | Risk of Bias in Non-randomized Studies of Interventions (ROBINS-I) | Moderate to high | Risk of selection bias from convenience sampling, potential confounding factors that were not controlled, and reliance on self-reported data. |
